# A Comprehensive Review on Harnessing Wearable Technology for Enhanced Depression Treatment

**DOI:** 10.7759/cureus.66173

**Published:** 2024-08-05

**Authors:** Pramod T Borghare, Disha A Methwani, Aniket G Pathade

**Affiliations:** 1 Otolaryngology, Mahatma Gandhi Ayurved College Hospital and Research, Wardha, IND; 2 Otolaryngology, NKP Salve Institute Of Medical Sciences & Research Centre And Lata Mangeshkar Hospital, Nagpur, IND; 3 Medicine, Jawaharlal Nehru Medical College, Datta Meghe Institute of Higher Education and Research, Wardha, IND

**Keywords:** telemedicine in mental health, personalized treatment, remote patient monitoring, mental health monitoring, wearable technology, depression treatment

## Abstract

Depression is a prevalent and debilitating mental health disorder that significantly impacts individuals, families, and societies worldwide. Despite advancements in treatment, challenges remain in effectively managing and monitoring depressive symptoms. Wearable technology, which encompasses devices that can monitor physiological and behavioral parameters in real time, offers promising new avenues for enhancing depression treatment. This comprehensive review explores the potential of wearable technology in managing and treating depression. It examines how wearables can monitor depressive symptoms, improve patient engagement and adherence to treatment plans, and provide valuable data for personalized treatment strategies. The review covers the integration of wearable technology in clinical settings, the role of wearables in remote monitoring and telemedicine, and the ethical and privacy considerations associated with their use. Additionally, it highlights case studies and pilot programs demonstrating the practical applications and outcomes of wearable technology interventions. Future directions and innovations are discussed, identifying potential advancements and challenges in this emerging field. This review aims to inform healthcare professionals, researchers, and policymakers about the opportunities and challenges of integrating wearable technology into depression treatment, ultimately contributing to improved mental healthcare outcomes.

## Introduction and background

Depression is a pervasive and debilitating mental health disorder that affects millions of individuals worldwide. It is characterized by persistent sadness, loss of interest or pleasure in daily activities, and various emotional and physical problems [1]. The World Health Organization (WHO) estimates that over 264 million people globally suffer from depression, making it a leading cause of disability. The burden of depression extends beyond the individual, impacting families, communities, and economies through lost productivity, increased healthcare costs, and reduced quality of life [2]. The impact of depression on global health is profound. It contributes significantly to the overall global burden of disease and is associated with various comorbid conditions such as anxiety, substance abuse, cardiovascular diseases, and diabetes [3]. Depression can lead to severe impairments in an individual's ability to function in social, occupational, and other important areas of life. In its most severe form, depression can result in suicide, with an estimated 800,000 people dying by suicide each year. This underscores the urgent need for effective and accessible treatments [4].

Wearable technology refers to electronic devices worn on the body as accessories or as part of clothing. These devices are equipped with sensors that collect data on various physiological and behavioral parameters, such as heart rate, sleep patterns, physical activity, and even emotional states. Wearables have become increasingly popular in recent years, driven by advances in sensor technology, data analytics, and connectivity [5]. Wearable technology has opened new avenues for health monitoring and intervention. Initially, wearables were primarily used for fitness tracking, but their applications have expanded significantly to include monitoring chronic diseases, managing mental health conditions, and providing personalized healthcare solutions. The integration of wearable technology into mental healthcare, particularly in the treatment of depression, represents a promising frontier in digital health [6]. This comprehensive review aims to explore the potential of wearable technology in enhancing the treatment of depression. It will examine how these devices can monitor and manage depressive symptoms, improve patient engagement and adherence to treatment plans, and provide valuable data for personalized treatment strategies. The review will also address the integration of wearable technology in clinical settings, the role of wearables in remote monitoring and telemedicine, and the ethical and privacy considerations associated with their use.

## Review

Understanding depression

Depression is a prevalent and serious mental health condition characterized by persistent sadness, loss of interest in activities, and a variety of emotional and physical issues [7]. The American Psychiatric Association identifies several types of depressive disorders. Major depressive disorder (MDD) involves at least one major depressive episode lasting two weeks or more, marked by significant impairment in daily functioning. Persistent depressive disorder, also known as dysthymia, is a chronic form of depression lasting at least two years, characterized by a low mood that, while less severe than MDD, can still be debilitating [7]. Other forms of depression include postpartum depression, which affects women after childbirth, featuring severe mood swings, exhaustion, and feelings of inadequacy. Bipolar depression occurs in individuals with bipolar disorder, where depressive episodes alternate with manic or hypomanic episodes. Seasonal affective disorder (SAD) is related to seasonal changes, typically worsening in the fall and winter months when daylight is reduced [8]. Atypical depression is characterized by mood reactivity (improvement in mood in response to positive events) and symptoms such as increased appetite and excessive sleep. Psychotic depression occurs when a major depressive episode includes psychotic features, such as hallucinations or delusions, often centered on themes of guilt or worthlessness [8]. Depression affects millions worldwide, with approximately 7% of adults in the United States experiencing depression annually and over 16% encountering it at some point in their lives [9]. The prevalence is notably higher among women and individuals assigned female at birth compared to men, and it also affects children, with about 4.4% of US children experiencing depression. Contributing factors to higher depression rates include genetic predisposition, environmental stressors, and co-occurring mental health conditions. Despite these statistics, many individuals do not seek help, resulting in underreporting and a significant treatment gap [10]. Treatment for depression usually involves a combination of psychotherapy, medication, and lifestyle changes. Common treatments include psychotherapy, such as cognitive behavioral therapy (CBT), which effectively addresses the underlying issues contributing to depression [11]. Medications, particularly selective serotonin reuptake inhibitors (SSRIs), are commonly prescribed; however, about one-third of patients may experience treatment-resistant depression, where standard treatments do not yield significant improvement. Lifestyle changes, including regular exercise, a healthy diet, and good sleep hygiene, are crucial in managing symptoms [11].

Wearable technology: an overview

Wearable technology encompasses a broad array of electronic devices designed to be worn on the body, offering functionalities that span health monitoring, fitness tracking, communication, and entertainment [12]. Often worn close to the skin, these devices collect and transmit data. Common examples include smartwatches (e.g., Apple Watch, Samsung Galaxy Watch), fitness trackers (e.g., Fitbit), smart glasses (e.g., Google Glass), virtual reality (VR) headsets (e.g., Oculus Rift), and smart clothing (e.g., garments with integrated sensors). These devices employ various technologies, such as microprocessors, sensors, and connectivity options like Bluetooth and Wi-Fi, to monitor and analyze user data in real time [12]. Wearable technology has diverse applications across multiple fields. In healthcare, wearables monitor vital signs, track medical conditions, and facilitate remote patient monitoring (RPM), improving healthcare management and reducing costs. In fitness and wellness, devices like fitness trackers provide insights into physical activity, sleep patterns, and overall health, helping users achieve fitness goals [13]. Entertainment applications include VR headsets that offer immersive gaming and media consumption experiences, enhancing user engagement through interactive environments. Fashion has also embraced wearable technology, with smart textiles and accessories combining style and functionality [13]. The advantages of wearable technology include real-time monitoring, allowing continuous tracking of health metrics and physical activity for immediate feedback and lifestyle adjustments [14]. Wearables offer convenience by providing easy access to information and notifications without the need to check smartphones, enhancing user connectivity and productivity. Some wearables include safety features like fall detection and location tracking, which can be crucial in emergencies. Additionally, many devices offer customizable features and styles, allowing users to express their individuality while benefiting from technology [14]. Despite their benefits, wearable technologies face challenges such as privacy and security concerns, as the collection of personal data raises issues regarding user privacy and data security. Battery life is another significant hurdle, as many devices require regular recharging. Integration and compatibility can also be complex, particularly in healthcare settings, where seamless integration with existing systems and devices is crucial [15]. As technology evolves, wearable devices are expected to become more sophisticated, leading to broader applications and enhanced user experiences. The market for wearables is projected to grow significantly, driven by advancements in artificial intelligence (AI), miniaturization, and connectivity [16].

Mechanisms of wearable technology in depression treatment

Wearable technology has emerged as a valuable tool in treating depression, leveraging various mechanisms to monitor physiological and behavioral indicators, provide biofeedback, and facilitate personalized treatment plans. One of the primary functions of these devices is to monitor physiological indicators such as heart rate, sleep patterns, and physical activity. For instance, heart rate variability (HRV) is a critical indicator of autonomic nervous system function and is often reduced in individuals with depression [17]. Wearables can measure HRV, offering insights into emotional regulation and stress levels. Additionally, wearables track resting heart rates, which can indicate heightened stress and anxiety, both of which are commonly associated with depression. Sleep disturbances are another hallmark of depression, and wearable devices can monitor sleep duration, quality, and cycles, helping users identify patterns that may exacerbate their symptoms. Furthermore, regular physical activity improves mood and reduces depressive symptoms, and wearables can encourage users to exercise by tracking their daily activity levels [17]. In addition to physiological monitoring, wearables can track behavioral patterns that provide insights into an individual's mental health. For example, some devices analyze communication patterns, such as the frequency and duration of social interactions [18]. Reduced social engagement is often a symptom of depression, and tracking these interactions can help identify periods of social withdrawal. Similarly, monitoring movement and activity levels can detect changes that may correlate with depressive episodes; a significant decrease in movement may indicate a worsening of symptoms [18].

**Figure 1:**
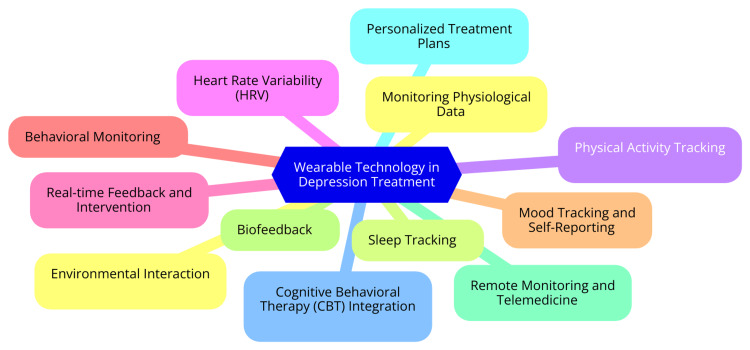
Mechanisms of wearable technology in depression treatment Image Credit: Dr. Aniket G. Pathade

Another important mechanism is biofeedback, a therapeutic technique that uses real-time data from wearables to help individuals gain awareness and control over their physiological functions. This approach can be particularly beneficial in managing depression. For instance, wearables can provide feedback on physiological responses to stress, such as heart rate and skin conductance [19]. By becoming aware of these responses, individuals can learn relaxation techniques, such as deep breathing or mindfulness, to manage stress effectively. Additionally, some devices offer guided interventions based on real-time data. If a user's heart rate indicates elevated stress, the device might prompt a breathing exercise or mindfulness session to help regulate mood [19]. The ability to collect and analyze data from wearables is crucial for developing personalized treatment plans. Continuous data collection from these devices provides a wealth of information over time, allowing for the identification of trends and patterns in mood, activity, and physiological responses. This continuous monitoring can highlight triggers and patterns that may not be evident in traditional clinical assessments [20]. Advanced algorithms can analyze the collected data to generate personalized insights and recommendations. For example, suppose a user shows a pattern of decreased activity leading up to depressive episodes. In that case, the system can suggest increasing physical activity or engaging in social activities during those times. Furthermore, data from wearables can be integrated into electronic health records (EHRs), providing clinicians with valuable information to inform treatment decisions. This integration enhances communication between patients and providers, fostering a collaborative approach to mental healthcare [20]. Mechanisms of wearable technology in depression treatment are shown in Figure 1.

Wearable technology in clinical settings

Integration of Wearable Devices in Clinical Practice

Integrating wearable devices into clinical practice represents a transformative shift in healthcare delivery, enabling more personalized and proactive patient care. However, this integration is not without its complexities. One of the foremost considerations is the need for clearly defined clinical problems that wearable technology can address [21]. For instance, if a healthcare provider aims to monitor patients with chronic conditions, the selected wearable device must be tailored to capture relevant physiological data to inform treatment decisions. Additionally, seamlessly integrating wearable data into EHRs and existing clinical workflows is essential. This ensures that healthcare providers can access and utilize the data effectively, enhancing patient management without complicating their routines [21]. Another critical aspect of successful integration is the emphasis on personalized experiences. Wearable technology should collect data and provide individualized feedback and interventions based on each patient's needs. This personalization enhances patient engagement and adherence to treatment plans [22]. Furthermore, alignment with reimbursement models is vital for the sustainability of these technologies in clinical settings. As healthcare systems increasingly adopt value-based care models, ensuring that wearable technology is recognized and reimbursed within these frameworks will be crucial for its long-term viability [22].

Case Studies and Pilot Programs

Several health systems have initiated pilot programs to explore integrating wearable devices into their clinical practices, yielding valuable insights into such initiatives' potential benefits and challenges. Companies like Overlap have developed technologies to enhance the integration of wearable data with EHRs, allowing for a more streamlined workflow for healthcare providers [23]. Similarly, Vivify Health has partnered with various health systems to tackle the challenges associated with the meaningful use of device data, focusing on improving patient engagement and clinical outcomes. Validic is another notable player in this space, enabling the integration of patient-generated data from wearables into EHRs, thus providing clinicians with a comprehensive view of their patients' health [23]. These case studies illustrate the promise of wearable technology in transforming patient care, particularly in chronic disease management and preventive health. However, they also highlight the hurdles that must be overcome to achieve successful integration. Issues such as data standardization, interoperability between devices, and the training of healthcare providers to utilize this technology effectively remain significant challenges that need to be addressed [15].

Effectiveness and Outcomes of Wearable Technology Interventions

The effectiveness of wearable technology interventions in clinical settings is an area of active research, and preliminary findings suggest various potential benefits. One of the most significant advantages is wearables' ability to improve patient care quality through continuous monitoring and real-time feedback. For instance, wearables can facilitate RPM, allowing healthcare providers to keep track of patient's health status outside of traditional clinical environments. This capability enhances patient engagement and enables timely interventions to prevent complications and hospitalizations [24]. Moreover, wearable devices can potentially reduce the overall cost of care by facilitating the early detection of health issues and promoting preventive measures. By leveraging data collected from wearables, healthcare providers can make informed clinical decisions that lead to better health outcomes and lower healthcare expenditures [25]. However, realizing the full potential of wearable technology in healthcare requires overcoming challenges related to user acceptance, data privacy and security, and managing large volumes of data. Addressing these challenges will ensure that wearable technology can be effectively integrated into clinical practice and deliver measurable improvements in patient outcomes [25].

Wearable Technology for Remote Monitoring and Telemedicine

Wearable technology is crucial in RPM by enabling continuous health data collection and real-time communication between patients and healthcare providers. These devices, which include smartwatches, fitness trackers, and specialized medical wearables, gather vital signs and other health-related metrics, such as heart rate, blood pressure, and oxygen saturation [26]. The data collected is transmitted wirelessly to healthcare professionals, allowing for timely interventions and better management of chronic conditions. This capability is particularly beneficial for patients with chronic illnesses, allowing for ongoing assessment without frequent in-person visits [26]. The benefits of wearables in RPM are significant. First, they contribute to improved patient outcomes by facilitating the early detection of health issues, enabling proactive care, and reducing the risk of severe complications. Second, wearables increase patient engagement by providing real-time feedback on health metrics, fostering a sense of control, and encouraging adherence to treatment plans [14]. Additionally, wearables can lead to cost-effective care by minimizing the need for in-person visits and hospital readmissions, ultimately lowering healthcare costs. Finally, wearables offer enhanced data accuracy, providing more consistent health data than traditional monitoring methods, which leads to better-informed clinical decisions [14].

Telemedicine, particularly when combined with wearable technology, presents several benefits for treating depression. One of the most significant advantages is accessibility; patients can receive mental health services from the comfort of their homes, which is especially beneficial for those in remote areas or with mobility issues [27]. Furthermore, real-time monitoring through wearables allows clinicians to track physiological indicators associated with depression, such as sleep patterns and physical activity, providing valuable data to tailor treatment plans effectively. Increased patient engagement is another benefit, as wearables can offer feedback and reminders that enhance adherence to therapy and medication regimens [27]. However, challenges remain in the implementation of telemedicine for depression treatment. Data privacy concerns are paramount, as transmitting sensitive health information raises questions about security and confidentiality [28]. Not all patients may have access to the necessary technology or possess the skills to use it effectively, which could widen health disparities. Lastly, while telemedicine offers convenience, it may lack the personal interaction that is often crucial in mental health treatment, potentially affecting the therapeutic relationship between patients and providers [28].

**Figure 2:**
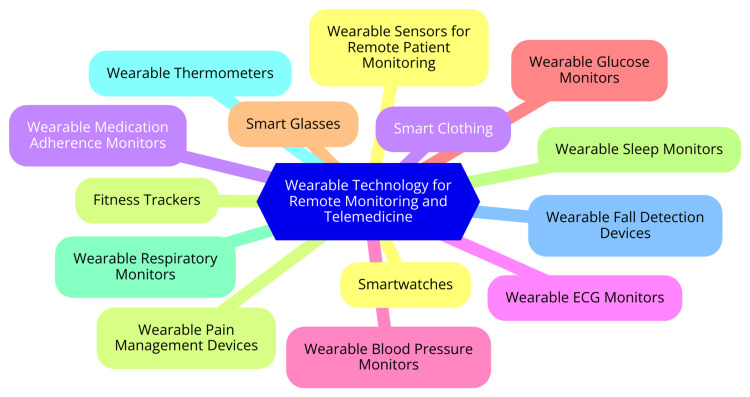
Wearable technology for remote monitoring and telemedicine Image Credit: Dr. Aniket G. Pathade

Several case studies highlight the successful integration of telemedicine and wearable technology in healthcare. One notable example is the VinCense platform, which utilizes clinical-grade wearables to monitor the health of elderly patients remotely [29]. This system has proven effective in managing chronic conditions by providing continuous health data to healthcare providers, allowing for timely interventions and improved treatment outcomes. Another example is the use of the Apple Watch in various studies, which monitors health parameters relevant to depression, such as HRV and activity levels. The data collected aids healthcare providers in understanding patients' mental health statuses and adjusting treatments accordingly [29]. Additionally, programs that incorporate wearables for chronic disease management, such as diabetes and hypertension, have demonstrated significant improvements in patient engagement and health outcomes. Continuous glucose monitors, for instance, provide real-time data that helps patients manage their conditions more effectively, reducing the need for emergency interventions [30]. These case studies illustrate the potential of wearable technology in enhancing remote monitoring and telemedicine, particularly in the context of mental health and chronic disease management. As technology continues to evolve, the integration of wearables into healthcare systems will likely expand, offering more personalized and efficient care solutions [30]. Wearable technology for remote monitoring and telemedicine is shown in Figure 2.

Enhancing patient engagement and adherence

Wearable technology has become a powerful tool for enhancing users' motivation, particularly in health and fitness contexts. The motivational mechanisms embedded within these devices are designed to foster engagement and promote healthier behaviors. One key aspect is real-time feedback, where wearables provide immediate feedback on physical activity, heart rate, and other health metrics. This instant gratification can reinforce positive behaviors and motivate users to maintain or increase their activity levels [31]. Another important factor is goal setting and tracking, as many wearable devices allow users to set personal health goals, such as step counts or weight loss targets. The ability to track progress toward these goals can enhance motivation and commitment to health regimens. Additionally, some wearables incorporate social features that allow users to share achievements with friends or participate in challenges. This social engagement can create community and accountability, motivating individuals to adhere to their fitness plans [31]. Gamification is a prevalent strategy in wearable technology that enhances user engagement by incorporating game-like elements into health and fitness activities. One approach includes points and rewards systems, where users earn points for completing tasks or reaching milestones, which can be redeemed for rewards [32]. This incentivizes continued use and adherence to health regimens. Many apps linked to wearables also offer challenges that encourage users to compete against friends or other users, which can drive higher engagement and motivation. Another effective strategy is progress visualization, where graphical representations of progress, such as charts and badges, help users visualize their achievements. This can enhance motivation by providing a tangible sense of accomplishment and encouraging users to push their limits [32]. The use of wearable technology has positively impacted adherence to treatment regimens, particularly in chronic disease management and fitness. Wearables help users become more aware of their health behaviors and outcomes, leading to better adherence to prescribed treatment plans, as users can see the direct effects of their actions. By providing data on health metrics, wearables enable users to self-regulate their behaviors, which is crucial for maintaining adherence to treatment regimens, as it encourages users to make informed decisions about their health [31]. The motivational aspects of wearables, combined with gamification strategies, support users in making long-term behavioral changes. Motivated and engaged users are likelier to adhere to their treatment regimens consistently. As these technologies evolve, their potential to transform health management and patient engagement continues to grow [31].

Ethical and privacy considerations

The integration of wearable technology in mental health treatment, particularly for conditions like depression, presents several significant privacy concerns and ethical implications that warrant careful consideration. One of the primary privacy concerns revolves around the data collected by these devices [33]. Wearables continuously gather sensitive personal health information, such as sleep patterns, physical activity levels, and physiological metrics like HRV. This data is often shared with third-party applications and services, sometimes without the explicit knowledge or consent of the user. Such practices raise alarms about who can access this sensitive information and how it may be used, leading to potential misuse or exploitation [33]. Moreover, the security of the data collected by wearable devices is critical. The vast amounts of personal data stored on these devices can be vulnerable to security breaches, resulting in identity theft, financial fraud, and reputational damage. Users may be unaware of the risks associated with data breaches, especially if manufacturers do not implement robust security measures [34]. Additionally, the lack of transparency regarding data collection practices further complicates the situation. Many wearable manufacturers do not communicate what data is collected, how it is used, or how long it is retained, making it challenging for users to make informed decisions about their privacy [34].

The ethical implications of constant monitoring through wearables are equally significant. These devices' ability to continuously monitor individuals' behaviors and health statuses can infringe upon personal privacy and autonomy [35]. Users may feel a sense of being constantly watched, which can lead to anxiety and discomfort. Informed consent is another crucial ethical consideration; participants in wearable technology studies must be fully aware of how their data will be collected, used, and shared. They should also have the right to withdraw from data collection without repercussions. Furthermore, the issue of equity and access cannot be overlooked. Not all individuals have equal access to wearable technology, which may exacerbate existing disparities in mental healthcare and treatment [35]. Robust regulatory frameworks and guidelines are essential to address these privacy and ethical concerns. Current regulations, such as the General Data Protection Regulation (GDPR) in Europe and the Health Insurance Portability and Accountability Act (HIPAA) in the United States, offer some protection for personal health data. However, these frameworks often fall short of addressing the unique challenges posed by wearable technology [36]. Industry self-regulation and voluntary codes of conduct are emerging as potential solutions, but there is a consensus that these measures may not adequately protect personal health data [36]. Ultimately, there is a pressing need for stronger regulations and enforcement mechanisms to ensure the responsible use and protection of personal health data collected by wearables. This includes implementing measures such as data encryption, establishing clear user consent protocols, and defining data ownership rights [37]. By fostering transparency and empowering users with control over their personal information, stakeholders can build trust in wearable technology and ensure its responsible deployment in mental health treatment. Addressing these concerns holistically will be crucial for maximizing the benefits of wearable devices while safeguarding user privacy and autonomy [37].

Future directions and innovations

The landscape of wearable technology for depression treatment is evolving rapidly, with significant advancements in sensors and data analytics. Wearable devices are increasingly being integrated into mental healthcare, allowing for real-time physiological and behavioral data monitoring. These devices can track metrics such as HRV, physical activity, and sleep patterns, critical indicators of mental health status [18]. For instance, studies indicate that increased physical activity correlates with lower depression rates, while HRV has been identified as a sensitive biomarker for depression. This ability to continuously monitor key indicators offers a new dimension to understanding and managing depression, leading to more timely and informed interventions [18]. Integrating AI and machine learning into wearable technology holds immense potential for enhancing data insights. AI algorithms can analyze large datasets collected from wearables, enabling early detection and personalized treatment strategies. Current research highlights that wearable AI has primarily been used for diagnostic purposes, with a notable gap in its application for treatment [38]. However, future advancements may focus on developing AI-driven interventions, such as personalized mindfulness practices or biofeedback therapy, which could be delivered through wearable devices. This would not only improve treatment accessibility but also allow for continuous monitoring and adjustment of therapeutic approaches based on real-time data. The potential to harness AI for predictive analytics could lead to proactive mental healthcare, where interventions are tailored to the individual's needs before symptoms escalate [38]. The integration of wearable technology with other treatment modalities presents a promising avenue for enhancing depression care. Combining wearables with telehealth services or mobile applications could facilitate a more holistic approach to mental health treatment. For example, wearables could provide continuous data to therapists, enabling them to tailor interventions based on objective measures of a patient's mental state [39]. Additionally, wearing wearables with CBT or other therapeutic techniques could enhance patient engagement and adherence to treatment plans. This synergistic approach empowers patients and allows healthcare providers to monitor progress more effectively [39].

Challenges and limitations

Wearable technology integrated with AI offers significant potential for enhancing depression treatment, but several challenges and limitations hinder its effective implementation and clinical adoption. One primary challenge is ensuring accurate and reliable data collection [40]. Current devices primarily gather data on physical activity, sleep patterns, and heart rate, which are crucial for assessing mental health conditions. However, integrating diverse data types, such as neuroimaging and self-reported measures, is necessary to enhance diagnostic accuracy and comprehensively understand a patient's condition. Developing algorithms capable of analyzing large volumes of complex data in real time remains a significant technical hurdle [40]. Moreover, while studies indicate promising accuracy rates for wearable AI in detecting depression, the technology is still in its early stages and not yet ready for widespread clinical use. Recent reviews show considerable variability in performance, highlighting the need for further research to improve reliability and validate findings across different populations and settings. Ensuring that wearable devices consistently provide accurate and clinically relevant data is essential for their acceptance and integration into mental health treatment [41]. Patient and clinician acceptance and trust are also crucial for the success of wearable technology in mental healthcare. Patients may be reluctant to adopt wearable devices due to concerns about privacy, data security, and the perceived value of the technology. Consistent use of the devices and accurate self-reporting of symptoms are critical for effective monitoring and intervention [42]. Comfort, ease of use, and perceived value significantly influence user compliance. Clinicians may be skeptical about the reliability and clinical relevance of data collected by wearable devices. Integrating these technologies into clinical practice requires building trust and demonstrating their value in improving patient outcomes. Clinicians must be confident in the accuracy of the data and the ability of wearable devices to provide meaningful insights into a patient's condition. Ongoing education and collaboration between technology developers and healthcare providers are essential for fostering acceptance and trust in wearable technology for depression treatment [42].

Cost and accessibility are additional significant barriers to the widespread adoption of wearable devices in mental health treatment. While consumer-grade wearables have become more affordable, specialized devices designed for clinical use may still be prohibitively expensive for many patients and healthcare systems [43]. Ensuring that wearable technology is accessible to diverse populations, regardless of socioeconomic status, is crucial for reducing health disparities and providing equitable access to mental healthcare. The availability of wearable devices may also vary by region and healthcare setting. Patients in underserved areas or those with limited access to specialized mental health services may face additional barriers in accessing wearable technology for depression treatment. Addressing these issues requires collaboration between technology companies, healthcare providers, and policymakers to develop strategies for increasing the availability and affordability of wearable devices in mental healthcare [43]. While wearable technology integrated with AI offers a promising approach to diagnosing and potentially treating depression, several challenges and limitations must be addressed to ensure its effective implementation and widespread adoption [44]. Improving device accuracy, fostering patient and clinician acceptance, and ensuring cost-effectiveness and accessibility are crucial steps in harnessing the full potential of wearable technology in mental healthcare. Ongoing research, collaboration, and innovation are essential for overcoming these barriers and providing accessible and effective depression treatment using wearable devices [44].

## Conclusions

Wearable technology represents a transformative advancement in treating and managing depression. By continuously monitoring physiological and behavioral indicators, wearables offer valuable insights that can lead to more personalized and effective treatment plans. The integration of wearable devices in clinical practice and telemedicine enhances patient engagement, adherence to treatment, and overall outcomes. Despite the promising potential, challenges such as data privacy, ethical concerns, and technical limitations need to be addressed to fully harness the benefits of this technology. Future innovations and research are essential to overcoming these obstacles and optimizing wearable technology for depression care. As the field evolves, wearable technology has the potential to significantly improve the quality of life for individuals with depression, offering a more proactive and holistic approach to mental health management.
